# Presence of antibacterial substances, nitrofuran metabolites and other chemicals in farmed pangasius and tilapia in Bangladesh: Probabilistic health risk assessment

**DOI:** 10.1016/j.toxrep.2021.01.007

**Published:** 2021-01-18

**Authors:** Md. Mehedi Alam, Mohammad Mahfujul Haque

**Affiliations:** a Department of Aquaculture, Bangladesh Agricultural University, Mymensingh, Bangladesh; b Department of Fishery Resources Conservation and Management, Khulna Agricultural University, Khulna, Bangladesh

**Keywords:** Aquaculture, Antibacterial substances, Heavy metals, Pesticides, Human health risks

## Abstract

Intensive feeding and the use of drugs and other chemicals for enhancing yield characterised commercial pangasius and tilapia aquaculture in Bangladesh. The residual presence of prohibited antibacterial substances, nitrofuran metabolites and other chemicals (ANCs) in fish, and their effect on public health are a concern for consumers. This study collected samples from 15 pangasius and 15 tilapia ponds to assess the contamination of ANCs, including pesticides, dyes and heavy metals in fish flesh, and heavy metals in feed, sediments and water. Antibacterial substances, nitrofuran metabolites and dyes in fish flesh were detected using LC–MS/MS. Organochlorine pesticides and heavy metals were detected applying GC–MS and AAS, respectively. We found very low residue of the most ANCs in pangasius and tilapia flesh, however, both species contained heavy metals, particularly lead (Pb) and chromium (Cr). The level of metal contamination was affected by the age of the pond; the highest concentration of Pb was in pangasius from old ponds (> 10 years), and the highest concentration of Cr was found in pangasius from new ponds (< 10 years), and tilapia from old ponds. The feed sampled in this study, particularly the commercial pellet and farm-made feed, were highly contaminated with heavy metals. Pond water and sediments were contaminated by heavy metals; fish, water and sediment samples from older ponds had higher concentrations of heavy metals. The concentration of these heavy metals in fish flesh above regulatory limits poses potential risks to human health. To ensure the production of safe fish for human consumption, commercial aquaculture in Bangladesh requires a functional regulatory framework.

## Highlights

• ANCs in pangasius and tilapia, fish feed, pond sediments and water were analysed comprehensively.
• Residual ANCs except heavy metals were not found in farmed pangasius and tilapia flesh.
• Residual toxic heavy metals lead and chromium in fish were above the permissible limit.
• Heavy metals sourced primarily from fish feed with secondary sources such as groundwater.
• Lead and chromium concentrations in fish flesh pose potential carcinogenic risks to human health.

## Graphical abstract

**Figure:**
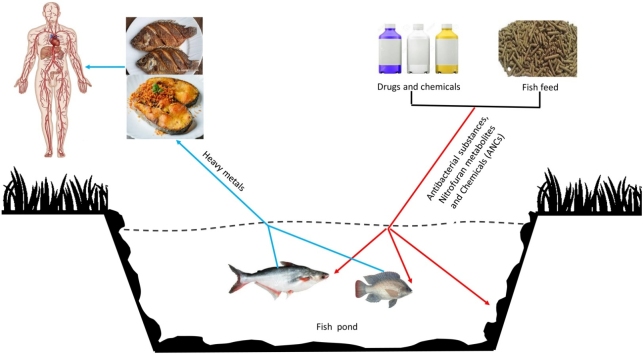

## 1 Introduction

The residual presence of antibiotics, heavy metals, pesticides and other chemicals in farmed fish is considered a significant risks to human health worldwide. Bangladesh has the fifth largest aquaculture production in the world, and is recognised as one of the leading countries for freshwater aquaculture [1]. Aquaculture dominates other animal food-producing sectors in terms of growth, and contributing 56 % of the country’s total fish production [2,3]. Fish act as the main source of animal protein in the diet of Bangladeshi people contributing a major portion (60 %) of animal protein intake [2]. The average fish consumptions in rural and urban area are 60.6 and 67.9 g day^−1^, respectively [4]. In Bangladesh, the sharp rise of aquaculture production started in the 1990s due to the development of intensive aquaculture technologies [3]. This intensive aquaculture is dominated by pangasius (*Pangasianodon hypophthalmus)* and tilapia (*Oreochromis niloticus*) farming, which collectively account for 36 % of the total aquaculture production in Bangladesh [2]. They are the primary farmed species, by volume of production, and characterised by cost-intensive farming for the investment for seed, feed, drugs, other chemicals, and labour that created a large domestic market with export potentials involving a large number of stakeholders across their value-chains. In farming of these species, the feed conversion ratio (FCR) is 2.0, and the cost of feed accounts for over 70 % of the total operating cost [3]. Previous studies have reported that feed is contaminated with various harmful substances, such as antibiotics, heavy metals, hormones, mycotoxins, organophosphates, anthelmintics and dyes [5,6].

With the expansion and intensification of aquaculture, farmers are increasingly facing problems of fish diseases, and the use of drugs and chemicals for treatment is increasing [7]. Pangasius and tilapia farming are affected by diseases, such as epizootic ulcerative syndrome (EUS), red spot disease (RSD), bacillary necrosis of pangasius (BNP), fin and tail rot, anal protrusion, pop-eye, gill rot, argulosis, and tilapia lake virus (TiLV) [8,9]. A study by Jahan et al. [10] found that fish mortality due to diseases cost US$80 – US$385 per ha of fish farm. A wide variety of water and sediment treatment compounds, fertilisers, pesticides, disinfectants, antibiotics, feed additives, hormones, vaccines, anaesthetics and probiotics are used to treat and control fish diseases, improve soil and water quality, and increase pond productivity [11]. Due to the absence of a functional aquaculture regulatory framework, there is a lack of technical advisory for the farmers, and therefore a lack of knowledge by the farmers, they use these materials unconcernedly [12]. The hazardous substances in the fish feed are directly consumed by the fish, while other substances used for soil, water and disease treatment mix with the water and precipitate in sediment, where they are absorbed by plankton and other food organisms. Ultimately most of the aquatic food organisms are consumed by the fish.

To produce safe fish, poultry and livestock products for domestic consumption and export trade, the Government of Bangladesh has sanctioned the Fish Feed and Animal Feed Act 2010, under the Ministry of Fisheries and Livestock (MoFL). To implement the act, the Department of Fisheries (DoF) has developed regulations for safe fish production as per Fish Feed Rules 2011 [13]. In these rules, a list of ANCs and the acceptable limits of their residues in fish feed and fish, has been outlined, in order to produce safe fish for human consumption. The key ANCs are prohibited antibacterial substances, nitrofuran metabolites, pesticides, dyes and heavy metals [13]. These ANCs are important food safety concerns for fish and fishery products according to European Union (EU) regulations, due to their adverse effects on human health [5,14]. The EU harmonised the legislation to control residues of ANCs, through the EU Council Regulation 2377/90/EC, and has set safe maximum residue limits in fish and fishery products being imported from Southeast Asian countries [15]. Due to the contamination of fish products by ANCs, particularly by chloramphenicol, nitrofuran metabolites, and malachite green, many consignments from Asian countries such as China, Thailand, Vietnam, and Bangladesh have been rejected several times by the EU [16]. This is because contamination of farmed fish by ANCs have severely detrimental effects on the humans who depend on farmed fish. The growing literature shows that the ingestion of ANCs through different contaminated foods can pose severe risks to human health. Rice consumed in Iran (including Iranian, Pakistani and Indian rice) had higher concentration of heavy metals than the national and international permissible limits, which pose unacceptable health risks, particularly non-carcinogenic risks to human health [17,18]. The high concentration of lead, cadmium and zinc in muscle of *Scomberomorous commerson* harvested from Persian Gulf was reported [19]. A systematic review conducted by Fakhri et al. [20] shows that the levels of arsenic and lead in the shrimp muscle were 1.37 and 0.58 ppm, respectively which were higher than safe limit recommended by FAO and other international agencies. Therefore, addressing ANCs is an important regulatory task for safe fish production in Bangladesh.

To our knowledge, there are no comprehensive studies on the presence of ANCs in farmed fish in Bangladesh. Very little is known, with regards to the chemicals and compounds found in fish flesh, in this context. Therefore, this research examines the presence of ANCs, such as antibiotics, antibacterial substances, organochlorine pesticides, heavy metals and dyes in commercially farmed fish flesh, in the context of the Fish Feed Rules 2011. In this study, the occurrence of ANCs in different sources, including feed, water and sediment in the aquaculture systems were systematically investigated. Our aim was to identify the ANCs, particularly those prohibited by MoFL and EU for use in aquaculture, in the flesh of pangasius and tilapia, as well as the possible sources of contamination, and their potential risks to the human health. This study aims to increase the existing information on contamination levels in these species, and provide a means to assist safe fish production for aquaculture for domestic consumption, as well as for export.

## 2 Materials and methods

### 2.1 Study area

**Fig. 1:**
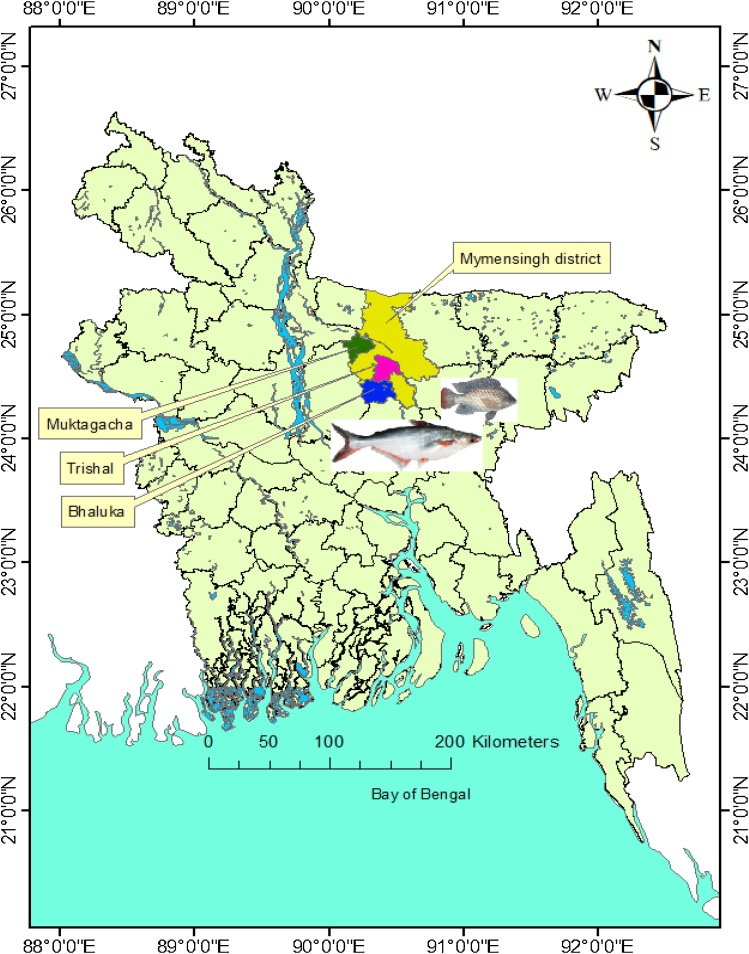
Map of Bangladesh showing the study sites in Mymensingh district.

The study area consists of three sub-districts (Upazila) in Mymensingh district of Bangladesh: Trishal, Bhaluka and Muktagacha (Fig. 1). Mymensingh is known as the ‘hub of aquaculture’, where commercial aquaculture was first developed in 1990 [21]. It is the top-ranked district for pond fish production in Bangladesh [2,3]. The selection of these study sites was justified based on the production data of pangasius and tilapia, as officially reported by the Department of Fisheries [2,3]. These three sites have had significant development of aquaculture due to suitable agroecological conditions. This includes the availability of ponds and agricultural land; a subtropical monsoon climate; fertile soil; and research and development support from Bangladesh Agricultural University (BAU), Bangladesh Fisheries Research Institute (BFRI), fish hatcheries, feed industries, and drug and chemical companies [3,10,21].

### 2.2 Study design

This study was designed to determine the residues of prohibited ANCs as per Fish Feed Rules 2011 [13] in aquacultured fish, pangasius and tilapia, and to identify the possible sources of contamination in the production chain, particularly in fish feed, pond sediment and water. A total of 30 ponds (15 pangasius and 15 tilapia) were selected; 10 in each sub-district. A simple structured questionnaire survey was carried out with these 30 fish farmers to gather data of their basic farming characteristics. We specifically selected ponds for sampling which had been commercially farming pangasius or tilapia for many years, used relevant feeding techniques, and with known use of drugs and chemicals. Older ponds tend to have a higher accumulation of contamination in the sediment and the water column and, therefore, in the flesh of fish. Consequently, ponds were categorised into two groups according to the time from construction: ponds at least 10 years old were considered old ponds, and those less than 10 years were considered new ponds (Table 1). The number of old and new ponds for pangasius and tilapia were eight and seven, and nine and six, respectively. From each pond, samples of fish, feed, sediment and water were collected to analyse the concentration of ANCs, such as antibacterial substances, nitrofuran metabolites, pesticides, heavy metals and dyes. Both pangasius and tilapia farmers applied two different types of feed, therefore, four different types of feed sample were collected for laboratory analysis to elucidate the differences in contamination among feed types (Table 1).

**Table 1:** Different types of sample collected for laboratory analysis to assess ANCs.

Type of samples	Total number of samples (N = 30)			
	No. of pangasius ponds (n = 15)		No. of tilapia ponds (n = 15)	
	Old	New	Old	New
Fish sample	8	7	9	6
Sediment sample	8	7	9	6
Water sample	8	7	9	6
Feed sample				
Farm-made feed	11	9		
Local brand sinking feed	4	6		
Commercial brand floating feed			8	9
Commercial brand sinking feed			7	6

### 2.3 Collection of fish samples

A total of 30 fish (15 pangasius and 15 tilapia) were randomly collected from 30 different ponds (i. e. one fish per pond) to achieve more reliable findings. The sampled pangasius were aged between 8 and 10 months and weighed between 1.0 and 1.2 kg; tilapia were aged between 3 and 4 months and weighed between 0.25 and 0.30 kg. These are typical values for this species as marketed in Bangladesh. According to the procedures of Rajeshkumar and Li [22], the sampled fish were washed with clean water at the point of collection, immediately placed on ice, and then frozen at −20 °C. Fish were dissected, the edible portion particularly flesh with skin and intermuscular bones was taken and rinsed with de-ionized water and frozen at −20 °C.

### 2.4 Collection of fish feed samples

Feeds used by the farmers were collected to determine the levels of heavy metal contamination as described by Kundu et al. [23]. Feed types consist of commercial brand^1^ floating and sinking feed, local brand^2^ sinking feed, and farm-made^3^ sinking feed. Pangasius farmers used local brand or farm-made sinking feed due to their lower cost and the long culture cycle of pangasius (∼12 months), compared to tilapia (∼4 months), requiring a significant investment in feed. Tilapia farmers used commercial floating feed for first two months, followed by commercial sinking feed. Tilapia has a short culture cycle, for this reason, farmers tried to maximise yield in a four-month period by using commercially manufactured, relatively expensive, floating and sinking feeds to get fish of marketable size.

### 2.5 Collection of pond sediment samples

Sediment from pangasius and tilapia ponds were collected to assess the levels of toxic heavy metals. Sediment samples were collected and analysed according to the procedures in [24,25] and Haque et al. [26]. An auger was applied to bore a hole from the pond bottom to a desired depth of between 10 cm–20 cm at three locations in each pond, and the three samples pooled. The sediment samples (n = 30) were stored in sealed polyethylene bags and kept on ice. They were then freeze-dried and passed through a 1 mm sieve to separate the stones, leaves, and dead invertebrates. The sediment was ground into a powder, using a mortar and pestle, and sieved through a 0.152 mm sieve (mesh no. 100), following the method of Adhikari et al. [27].

### 2.6 Collection of water samples

To analyse heavy metal concentrations, the water samples were collected just below the surface at two different locations per pond. The water samples were collected at three time points during the culture period; during fish stocking, in the middle of culture period, and at harvesting time, and the results were averaged. Before analysis of heavy metals, the samples were processed according to the standard procedure, as described by Rajput et al. [28] and Bridgewater et al. [29].

### 2.7 Analysis of samples

Samples were analysed in the laboratory of Fish Inspection and Quality Control (FICQ) at the Department of Fisheries (DoF), Chittagong, accredited according to ISO 17025: 2005 by the Bangladesh Accreditation Board (BAB). According to EU requirements for safe fish production and trade [16,30], the residues of ANCs, such as antibacterial substances (chloramphenicol, tetracycline, oxytetracycline and chlortetracycline), nitrofuran metabolites (furazolidon metabolite (AOZ), furaltadon metabolite (AMOZ), nitrofurantoin metabolite (AHD) and nitrofurazon metabolite (SEM)), organochlorine pesticides (DDT, aldrin, dieldrin, endrin and heptachlor), dyes (malachite green, leuco-malachite green, crystal violet, leuco-crystal violet) and heavy metals (lead, cadmium, mercury and chromium) in the fish body were tested. Residues of antibacterial substances, nitrofuran metabolites and dyes in fish flesh were detected using liquid chromatography – mass spectrometry (LC–MS/MS). The quantification of tetracyclines (tetracycline, oxytetracycline, and chlortetracycline) was based on the method described by Xu et al. [31]. For quantifying chloramphenicol and nitrofuran metabolites, the method in US FDA/CFSAN [32] was followed. Organochlorine pesticides were quantitatively analysed using gas chromatography – mass spectrometry (GC–MS) (using a Hewlett-Packard (HP) 6890 N coupled with a HP-5973 mass selective detector (MSD) and a 30 m ×0.25 mm ×0.25 μm DB-5 capillary column; J & W Scientific Co. Ltd., USA). The procedures for extraction and cleanup were based on those in Wang et al. [33]. In the FIQC laboratory, the heavy metals in fish flesh were analysed using an atomic absorption spectrophotometer (AAS) (ZEEnit-700 P) calibrated with standard solutions (Merck, Germany) [34].

Heavy metals in fish feed, pond sediments and water samples were analysed in the laboratory of the Interdisciplinary Institute for Food Security (IIFS) in BAU. The concentrations of heavy metals were examined with an AAS (Perkin Elmer, Model 1025) using a hollow-cathode lamp, following the standard procedures of Karadede & Unlu [35]. The wavelengths used for mercury (Hg), cadmium (Cd), lead (Pb), chromium (Cr), zinc (Zn), and arsenic (As) measurement were 253.7, 228.8, 283.3, 357.9, 213.9, and 193.7 nm, respectively. A standard reference material [dogfish liver (DOLT-2); CNRC, Canada] was used to verify the accuracy of the heavy metal concentrations, as described by Yap et al. [36].

### 2.8 Heavy metals exposure estimation and risk characterisation methodology

To assess the potential risks of heavy metals on human health from the tested fishes, the estimated daily intake (EDI), the target hazard quotients (THQs), and carcinogenic risk ratio (R) were used in the health risk assessment as introduced by Chien et al. [37], and followed by Cui et al. [38] and Sharafi et al. [17]. The EDIs for the analysed heavy metals were calculated by multiplying the average detected concentration (Table 3) in composite fish samples, by the weight of fish consumed by an average individual in Bangladesh (from the ‘Report on the household income and expenditure survey 2016’ [4]). A THQ < 1 denotes that the daily exposure causes no deleterious effects to human health. The acceptable level of R ranges from 1 × 10^−4^ (risk of developing cancer over a human lifetime is 1 in 10000) to 1 × 10^-6^ (risk of developing cancer over a human lifetime is 1 in 1000000) as described by [37] and [39]. In general, an R < 10^-6^ is considered acceptable for developing cancer, an R > 10^-4^ is unacceptable, and values between 10^-4^ and 10^-6^ are generally considered potential for cancer development in humans [40]. THQ and R values were determined in pursuance of the methods described by Chien et al. [37], Sharafi et al. [17] and Yu et al. [39], respectively. The formulae for calculating of EDI, THQ and R are in the Eqs. (1), (2), (3) as follows:

(1) $$\text{EDI}=\frac{\text{C}\;\times {\text{W}}_{\text{F}}}{{\text{W}}_{\text{A}\text{B}}}$$

where, C = pollutant concentration in food (μg g^−1^), W_F_ = daily average consumption of fish in the study area (according to BBS [4], assuming 62.58 g day^−1^ person^−1^), and W_AB_ = average body weight (70 kg for an adult).

(2) $$\text{THQ}=\frac{{\text{E}}_{F}\;\times {\text{E}}_{\text{D}}\times F_{\mathrm{IR}}\times C}{{\text{R}}_{\mathrm{FD}}\;\times {\text{W}}_{\text{A}\text{B}}\times T_{A}}\times 10^{-3}$$

(3) $$\text{R}=\frac{{\text{E}}_{\text{F}}\;\times {\text{E}}_{\text{D}}\times \text{S}\text{F}\times \text{C}}{{\text{W}}_{\text{A}\text{B}}\times {\text{T}}_{\text{A}}}\times 10^{-3}$$

where, E_F_ = exposure frequency (350 days year^−1^); E_D_ = exposure duration (70 years); F_IR_ = food ingestion rate (g person^−1^ day^−1^), R_FD_ = oral reference dose (mg kg^−1^ day^−1^ (body weight)), T_A_ = average exposure time (365 days year^−1^× lifetime, assuming 70 years), and SF = oral cancer slope factor (mg kg^−1^ day^−1^)^−1^. In this study, the concentrations used in the health risk calculations were all on a wet weight basis. A risk-based concentration table for individual heavy metals was used to look for R_FD_ and SF values [41].

Sometimes, the carcinogenic risk from exposure to a single toxicant might be low, but there are several heavy metals that pose a risk to human health. When toxicants number is two or more, some additives and/or their interactive effects may increase the carcinogenic risk to human health [42]. The summation of the non-carcinogenic risks is the hazard index (HI), and the total carcinogenic risks is R_T_. The calculations of HI and R_T_ were done following the formulas (4) and (5) from Yu et al. [39]:

(4) $$\text{H}\text{I}=\sum_{\text{i}=1}^{\text{m}}\text{T}{\text{H}\text{Q}}_{\text{i}}\;$$

(5) $${\text{R}}_{\text{T}}=\sum_{\text{i}=1}^{\text{m}}{\text{R}}_{\text{i}}\;$$

This assumes that the available contaminant concentration in humans is not altered by the food preparation or cooking process and the ingested pollutants are absorbed completely by the consumer.

### 2.9 Data analysis

A brief descriptive statistical analysis was carried out to explain the general background of pangasius and tilapia farming. A one-way analysis of variance (ANOVA) with a Duncan’s multiple range test (DMRT) was used to compare different parameters of the four types of pond, and the feed samples (values of p < 0.05 were taken to be statistically significant). An independent sample T-test was conducted to compare the parameters between pangasius and tilapia feeds. All the statistical tests were done using the statistical software SPSS (Statistical Package for Social Science) version 20 (IBM SPSS, Armonk, NY, USA).

## 3 Results

### 3.1 General characteristics

Descriptive statistics are summarised in Table 2, which presents the characteristics of the 15 pangasius and 15 tilapia farms in this study. On average, the pangasius farms and ponds were larger than those of the tilapia: pond size ranged from 0.16 ha (tilapia old ponds) to 0.91 ha (pangasius old ponds). Pond depth ranged from 1.31 m (tilapia new ponds) to 2.33 m (pangasius old ponds). New ponds had better installation of inlet and outlet systems compared to old ponds. Most farmers had a borehole pump used for irrigating their ponds with underground water. In old farms, sludge removal occurred every 3–4 years, while in new ponds it happened more frequently (every 1–2 years).

**Table 2:** General characteristics of pangasius and tilapia farms.

Criteria/Indicators	Pangasius pond (*n = 15*)		Tilapia pond (*n = 15*)	
	Old (*n = 8*)	New (*n = 7*)	Old (*n = 9*)	New (*n = 6*)
Farm characteristics				
Farm size (ha)	2.90 ± 1.37	1.07 ± 0.23	1.89 ± 1.34	0.81 ± 0.12
Number of ponds per farm	6.3 ± 4.3	4.3 ± 1.3	7.7 ± 5.2	3.2 ± 0.0
Research pond characteristics				
Pond size (ha)	0.91 ± 0.37	0.23 ± 0.02	0.16 ± 0.05	0.18 ± 0.04
Age of pond (year)	16.7 ± 4.3	5.2 ± 1.3	14.3 ± 13.4	3.7 ± 1.2
Water depth (m)	1.92 ± 0.58	2.23 ± 0.55	1.83 ± 0.46	1.31 ± 0.09
Inlet-outlet system (%)	58	79	67	86
Water source (%) Underground water Rain water	74 26	81 19	78 22	75 25
Interval of sediment removal (year)	4.3 ± 0.6	1.7 ± 0.3	3.1 ± 0.0	1.4 ± 0.3
Use of drugs and chemicals (% farmers)				
Water and soil treatment compounds	100	100	100	100
Fertilisers	29	37	18	13
Disinfectants	11	7	8	13
Antibiotics	17	11	15	9
Pesticides	12	6	5	2
Probiotics	21	14	8	3
Feed additives	32	21	26	36
Investments for drugs and chemicals (US$ ha^−1^)	208	158	135	112
Used of feed type (% farmers) Farm-made sinking Local brand sinking Commercial brand floating Commercial brand sinking	73 27 0 0	60 40 0 0	0 0 53 47	0 0 60 40

**Table 3:** Presence of antibacterial substances, nitrofuran metabolites and other chemicals (ANCs) in fish flesh.

ANCs	Residues of ANCs	Acceptable limit (set by EU)	Pangasius ponds (*n = 15*)		Tilapia ponds (*n = 15*)	
			Old (*n = 8*)	New (*n = 7*)	Old (*n = 9*)	New (*n = 6*)
Antibacterial substances	Chloramphenicol	0.0 ppb	0	0	0	0
	Tetracycline	100 ppb	<100	<100	<100	<100
	Oxytetracycline	100 ppb	<100	<100	<100	<100
	Chlortetracycline	100 ppb	<100	<100	<100	<100
Nitrofuran metabolites	Furazolidon metabolite (AOZ)	0.0 ppb	0	0	0	0
	Furaltadon metabolite (AMOZ)	0.0 ppb	0	0	0	0
	Nitrofurantoin metabolite (AHD)	0.0 ppb	0	0	0	0
	Nitrofurazon metabolite (SEM)	0.0 ppb	0	0	0	0
Organochlorine pesticides	Aldrin	0.005 ppm	NF	NF	NF	NF
	DDT	1.00 ppm	NF	NF	NF	NF
	Heptachlor	0.005 ppm	NF	NF	NF	NF
	Dieldrin	0.005 ppm	NF	NF	NF	NF
	Endrin	0.01 ppm	NF	NF	NF	NF
Dyes	Malachite green	0.0 ppb	0	0	0	0
	Leuco-malachite green	0.0 ppb	0	0	0	0
	Crystal violet	0.0 ppb	0	0	0	0
	Leuco-crystal violet	0.0 ppb	0	0	0	0
Heavy metals	Lead (Pb)	0.30 ppm	1.95 ± 0.03	1.30 ± 0.21	1.35 ± 0.19	1.34 ± 0.08
	Cadmium (Cd)	0.05 ppm	0.01	<0.005	0.01	<0.005
	Mercury (Hg)	0.50 ppm	NF	NF	NF	NF
	Chromium (Cr)	0.01 ppm	<0.09	0.54 ± 0.02	0.20 ± 0.01	<0.09

NF = Not found.

Water and soil treatment compounds, fertilisers, disinfectants, antibiotics, pesticides, probiotics, and feed additives were applied by farmers to treat the water, sediment and fish diseases. The average per hectare investment for drugs and chemicals ranged from US$ 112.0 to US$ 208.0, with pangasius old ponds incurring the highest costs. Pangasius farmers used farm-made feed in 73 % of old and 60 % of new ponds, and the remaining 27 % and 40 % used local brand sinking feed. All tilapia farmers used commercial brand feeds, of which floating feed was used slightly more frequently in both old and new ponds (53 % and 60 %, respectively).

### 3.2 Presence of ANCs in fish flesh

The residual presence of ANCs in fish flesh are presented in Table 3. As per the guidelines outlined in the Fish Feed Rules 2011 [13], the recommended acceptable concentrations in fish flesh are shown as reference values. These results show that concentrations of antibacterial substances were below acceptable limits, in both pangasius and tilapia. Nitrofuran metabolites, organochlorine pesticides and dyes were completely absent.

The concentrations of the heavy metals Pb, Cd, Hg and Cr in pangasius and tilapia flesh are presented in Table 3. There was no significant difference (p > 0.05) between different types of pond in concentration of Pb. However, irrespective of pond age, Pb concentrations were between five and seven times higher than the acceptable limit (0.30 ppm): pangasius from old ponds contained the highest Pb concentrations (1.95 ppm), compared to new ponds (1.30 ppm) without significant difference between them. In tilapia, Pb concentrations were equivalent in both old and new ponds. Cd concentrations were below the acceptable limit in all ponds, but slightly higher in old ponds (NS, p > 0.05). Hg was not present in any of the samples. Concentrations of Cr varied significantly (p < 0.05) depending on pond type and fish species. The highest concentration of Cr was found in pangasius from new ponds (0.54 ppm), followed by tilapia in old ponds (0.20 ppm), both of which exceed the recommended limit of 0.01 ppm. In pangasius from old ponds and tilapia from new ponds, Cr concentrations were below the acceptable limit (<0.09 ppm). Overall, the relative concentrations of heavy metals in the fish flesh analysed in this study are: Cr > Pb > Cd > Hg.

### 3.3 Presence of heavy metals in fish feed samples

Since heavy metals were detected in fish flesh, feed samples were tested to identify the source of contamination. Table 4 shows that, with the exception of Hg, heavy metals (Cd, Pb, Cr, Zn and As) were found in all sampled feeds. Cd concentrations ranged between 0.25 ppm to 0.33 ppm; five to seven times higher than the acceptable limit (0.05 ppm). The highest Cd concentration was found in local brand sinking feed (0.33 ppm), but there was no significant difference between the types of feed (p > 0.05). As with the contamination of fish, all types of fish feed were highly contaminated with Pb and Cr, with no significant difference (NS, p > 0.05) between the feed types. Concentrations of Pb and Cr ranged from 0.92 ppm to 4.47 ppm, and 0.93 ppm–16.34 ppm, respectively. The farm-made sinking feed contained the highest levels of both Pb and Cr. Concentrations of Zn and As were also found above acceptable limits in all and two types of feed, respectively.

**Table 4:** Presence of heavy metals in different types of fish feed (Mean ± SE).

Feed type	Brand	Hg (ppm)	Cd (ppm)	Pb (ppm)	Cr (ppm)	Zn (ppm)	As (ppm)
Acceptable limit (set by EU)		0.50	0.05	0.30	0.10	50.00	1.00
Floating	Commercial	0.0 ± 0.0	0.25 ± 0.12	0.92 ± 0.26	1.35 ± 0.45	76.02 ± 2.3	0.82 ± 0.20
Sinking	Commercial	0.0 ± 0.0	0.30 ± 0.17	1.49 ± 0.25	0.93 ± 0.18	81.77 ± 2.1	1.24 ± 0.39
Sinking	Local	0.0 ± 0.0	0.33 ± 0.07	1.82 ± 0.45	4.43 ± 1.42	64.16 ± 1.1	2.67 ± 0.73
Sinking	Farm-made	0.0 ± 0.0	0.26 ± 0.10	4.47 ± 1.12	16.34 ± 2.7	72.63 ± 1.9	≤0.20
*p* value		–	0.506	0.093	0.271	0.144	0.361

### 3.4 Presence of heavy metals in pond sediments

As with the fish feed, our analyses show that, with the exception of Hg, pond sediment was contaminated with heavy metals (Cd, Pb, Cr, Zn and As) (Table 5). Levels of Cd ranged from 0.20 ppm to 0.27 ppm in sediments from all ponds (NS, p > 0.05). Concentrations of Pb were higher in pangasius ponds (old and new) than in tilapia ponds (NS, p > 0.05). Concentrations of Cr were highest in sediment from old pangasius ponds (31.13 ppm) and lowest in sediment from new tilapia ponds (10.30 ppm). Sediment from tilapia new ponds had significantly lower levels of Cr (p < 0.05). There was no significant difference in concentrations of Zn between the types of pond (range: 72 ppm to 75 ppm). Concentrations of As were significantly higher in pangasius pond sediments (p < 0.05), and were higher in old pangasius ponds (NS, p > 0.05; range: 31 ppm to 43 ppm).

**Table 5:** Presence of heavy metals in pond sediment and water of pangasius and tilapia ponds (Mean ± SE).

Heavy metals	Pangasius ponds (*n = 15*)		Tilapia ponds (*n = 15*)		*p* value
	Old (*n = 8*)	New (*n = 7*)	Old (*n = 9*)	New (*n = 6*)	
Presence of heavy metals in pond sludge/sediment					
Hg (ppm)	0.0 ± 0.0	0.0 ± 0.0	0.0 ± 0.0	0.0 ± 0.0	–
Cd (ppb)	0.25 ± 0.12	0.22 ± 0.06	0.20 ± 0.05	0.27 ± 0.08	0.997
Pb (ppm)	27.21 ± 3.22	25.93 ± 6.09	20.69 ± 2.47	17. 97 ± 1.06	0.376
Cr(ppm)	31.13 ± 6.11^a^*	23.39 ± 10.39^a^	18.04 ± 6.95^a^	10.30 ± 0.57^b^	0.038
Zn (ppm)	75.03 ± 8.20	72.98 ± 6.09	72.33 ± 5.13	75.67 ± 5.01	0.230
As (ppm)	43.67 ± 11.54^a^	42.28 ± 7.57^a^	33.13 ± 5.37^b^	31.74 ± 5.23^b^	0.052
Presence of heavy metals in the water					
Hg (ppm)	NF	NF	NF	NF	–
Cd (ppb)	0.01 ± 0.00	NF	NF	NF	–
Pb (ppm)	0.27 ± 0.05^b^*	0.09 ± 0.19^a^	0.04 ± 0.01^a^	0.03 ± 0.01^a^	0.055
Cr (ppm)	0.05 ± 0.01^b^	0.37 ± 0.02^a^	0.13 ± 0.01^b^	0.07 ± 0.01^b^	0.038
Zn (ppm)	0.03 ± 0.01	0.02 ± 0.00	0.02 ± 0.00	0.01 ± 0.00	0.729
As (ppm)	0.07 ± 0.01^a^	0.09 ± 0.02^a^	0.18 ± 0.03^b^	0.04 ± 0.01^a^	0.024

*Figures with similar superscripts in a row do not indicate significant difference (p<0.05). NF = Not found.

### 3.5 Presence of heavy metals in pond water

Table 5 also shows the concentrations of heavy metals in the water. Hg was not found, and Cd was only found in old pangasius ponds in minimal amounts (<0.005 ppm to 0.01 ppm). Concentrations of Pb were significantly higher in the water from pangasius old ponds (0.27 ppm, p < 0.05) (between 0.03 ppm and 0.27 ppm). Water from pangasius new ponds had the highest level of Cr compared to other ponds (p < 0.05) (between 0.05 ppm and 0.37 ppm). There were no significant differences in Zn concentrations (between 0.01 ppm and 0.03 ppm). Arsenic was found at significantly higher concentrations in tilapia old ponds, compared to other ponds (p < 0.05; range: 0.04 ppm to 0.18 ppm).

### 3.6 Human health risks of heavy metals due to fish consumption

Table 6 compares our results with the criteria issued by regulatory agencies. All the values in the present study were less than the regulatory standards, with the exception of Cr in pangasius flesh, which had an EDI of 0.283 mg d^−1^, slightly higher than the recommended limit (0.20 mg d^−1^). The relative THQ of the targeted heavy metals, from highest to lowest, are Pd > Hg > Cr > Cd. All heavy metals had a THQ < 1.0. The HI (total THQ) did not exceed 1; collective non-carcinogenic risks were 0.491 for pangasius and 0.370 for tilapia. The R values for individual Pb, Cd and Cr were below the acceptable range between 1 × 10^-4^ to 1 × 10^-6^. The R_T_ (total R) was 2.42 × 10^-6^ and 1.15 × 10^-6^ for pangasius and tilapia flesh, respectively; within the acceptable limits for potential carcinogenic risk in humans.

**Table 6:** The estimated daily intake (EDI), non-carcinogenic (THQ) and carcinogenic (R) risks of studied contaminants.

Heavy metals	EDI			THQ			R		
	Pangasius	Tilapia	MTDI (mg/kg bw/day)	Pangasius	Tilapia	R_FD_ (mg/kg/day)	Pangasius	Tilapia	SF (mg/kg/day)^−1^
Pb	1.453	1.204	3.0 mg d^−1^ [43]	0.164	0.136	0.004 [38]	1.89 × 10^−7^	1.57 × 10^−7^	0.0085 [44]
Cd	0.007	0.006	0.5 mg d^−1^ [45]	0.006	0.005	0.001 [38]	4.16 × 10^−8^	3.64 × 10^−8^	0.38 [46]
Hg	0.023	0.019	0.03 mg d^−1^ [47]	0.231	0.188	0.0001 [38]	—	—	—
Cr	0.283	0.128	0.20 mg d^−1^ [48]	0.090	0.041	0.003 [38]	2.19 × 10^−6^	9.58 × 10^−7^	0.5 [38]
HI and R_T_	—	—		0.491	0.370		2.42 × 10^−6^	1.15 × 10^−6^	

MTDI = Maximum Tolerable Daily Intake; R_FD_ = Oral reference dose; SF = Oral cancer slope factor; 1 mg/kg = 1 ppm.

## 4 Discussion

### 4.1 General characteristics

Pangasius and tilapia farming largely depend on groundwater irrigation pumps [3] particularly during the dry season (February to May), which is a possible cause of contamination of pond water and sediments with heavy metals. Groundwater has been reported to contain heavy metals at concentrations which often exceed the guideline values for Bangladesh, as recommended by WHO [49]. Sediment removal occurs less frequently in older ponds than in newer ponds, and in pangasius farms compared to tilapia farms. This leads to the deposition of large volumes of sludge and nutrients in the benthic sediments [26]. Pangasius and tilapia farmers use different categories of drugs and chemicals for soil and water treatment in order to increase primary productivity, treat diseases and enhance the growth of cultured fish. All the farmers in this study used different chemicals for water treatment which are lime, salt, zeolite and potassium permanganate. Lime contains heavy metal impurities such as Cd, which can accumulate in the sediments, be taken up by fish, and passed up the food chain to humans [50]. Other chemicals such as fertilisers, disinfectants, antibiotics, pesticides, probiotics and feed additives were used by a small proportion of farmers. The use of these chemicals suggests that in commercial aquaculture, maintaining water and soil quality parameters is one of the key management practices. These water treatment chemicals may contain heavy metals and other contaminants that can leach into water or sediments where they can be absorbed by fish. Pangasius farmers tended to use farm-made sinking pellet feeds as the price of these are considerably lower than commercial feeds. On the other hand, tilapia farmers predominantly used commercial brand floating and sinking feed throughout the production cycle. The feed ingredients and feeds purchased from local markets vary in quality, which could be a potential source of contamination [51].

### 4.2 Bioaccumulation of ANCs in fish flesh

#### 4.2.1 Contamination levels in fish flesh

The presence of antibacterial substances and nitrofuran metabolites in farmed fish, is a major human health concern in domestic and export market of fish. The antibacterial substances and nitrofuran metabolites were not found in pangasius and tilapia flesh in this study, suggesting farmers deliberately did not use them in their farms. Between 2017 and 2018, the DoF analysed more than 50 samples of pangasius and tilapia collected from the Mymensingh region and found no traces of prohibited chloramphenicol and nitrofuran in fish flesh [52]. The residues of organochlorine pesticides cause harmful effects in the liver, lungs, kidneys, thyroid, reproductive tissues, and nervous and immune systems of human being [53]. In our study, organochlorine pesticides were not found in either pangasius or tilapia flesh, indicating the fish in this study are cultured in systems that are free from these chemicals. Heavy metal residues in fish can be toxic for human health: Pb is a non-essential element that can cause a range of adverse effects such as neuro and nephrotoxicity; rapid behavioral malfunction; decreased growth, metabolism, and survival rate; and modifications to social behavior [18]. It is toxic and damages the brain, kidneys and reproductive systems of humans [54,55]. In our study, Pb contamination was detected in both pangasius and tilapia flesh; fishes in new ponds were less contaminated than those produced in old ponds. According to the US FDA and EU regulations for farmed fish in Bangladesh, the permissible limit for Pb is 0.30 ppm. In our study, all the fish samples contained Pb (>1.30 ppm) in concentrations above the permissible limit. A previous study in Bangladesh done by Ullah et al. [44] showed that the concentrations of Pb in pangasius (0.947 ppm) and tilapia (0.313 ppm) flesh were above the permissible limit for human consumption. Lead concentration in muscle of *Scomberomorous commerson* ranged from 1.43 to 2.02 ppm in Iran [19]. Findings of the previous studies are consistent with the results of our study, suggesting that fish may contain higher concentration of Pb which poses risks to human health.

The accumulation of Cd in humans can induce skeletal damage, and kidney and reproductive system dysfunction [17]. In this study, Cd was found in the flesh of both pangasius and tilapia farmed in old ponds, at a concentration of 0.01 ppm, well below the recommended limit of 0.05 ppm. Hg is highly toxic for animals, and has deleterious effects on the nervous, digestive and immune systems, and on lungs, kidneys, skin and eyes. Hg was not found in any of the analyses in this study. Cr accumulation is of great concern because, even in small quantities, it is a toxic substance which has no known biological function in the human body. The concentrations of Cr in analysed fish flesh were 54 and 20 times higher than the recommended levels, in pangasius old ponds and tilapia new ponds, respectively. Ullah et al. [44] reported that the residues of Cr in pangasius (0.121 ppm) and tilapia (0.086 ppm) flesh were higher than the legislative values for human consumption. Similarly, a study by Ahmed et al. [56] showed that residues Cr in pangasius (1.349 ppm) and tilapia (1.274 ppm) flesh were above the permissible limits. Compared with the previous studies, our study indicates that consumption of farmed pangasius and tilapia is a food safety concern due to the high concentrations of Cr.

#### 4.2.2 Potential source of bioaccumulation of heavy metals

According to Rajeshkumar and Li [22], there are two main ways heavy metals can bioaccumulate in fish; by direct consumption of water and feed through the digestive tract, and non-dietary routes across permeable membranes, such as muscle and gills. Fatema et al. [57] reported the accumulation of heavy metals such as Pb, Cd, and Cr from commercial fish feeds in Bangladesh caused higher concentrations of heavy metal in fish than the permissible limits set by the FAO and WHO. The results of the present study revealed that almost all types of fish feed used in pangasius and tilapia farms were contaminated with heavy metals above the permissible limit. Botaro et al. [58] reported a positive correlation between total heavy metal concentrations in fish muscle and the concentrations in the supplied feeds. Pangasius farmers tend to prepare fish feed in their own farms using a variety of ingredients. In particular, meat and bone meal originated from land-based animals are used as the main source of protein and is bought from suppliers who import it internationally. Studies have shown that meat and bone meal contain heavy metals which originate from the processing plants, while slaughterhouse by-products are processed to produce the feed ingredients [59]. Other studies have reported that feed processors mix the low-cost tannery wastes, containing heavy metals, with other feed ingredients to produce metal-contaminated fish feed [60]. Farmers are unlikely to have access to information regarding the quality of feed ingredients from suppliers. Commercial feeds for tilapia are reported to contain heavy metals, with particularly high concentrations of Cr [61], similar to the results found in this study. Although feed rules and regulations have been developed by the government, poor implementation of the fish feed rules [13] and inadequate enforcing of regulations and feed standards are responsible for the contamination of fish feed in Bangladesh [62].

Fish have the ability to accumulate heavy metals in their tissues from the sediment and water of their aquatic environment. Sediment is the major depository of metals, absorbing and storing more than 99 % of the total amount of metals that are present in the aquatic system [63]. The main sources for elevated levels of heavy metals in pond sediments are uneaten fish feed, faeces, fertilisers, pesticides, animal and poultry manures, groundwater, wastewater irrigation and agricultural discharges [24,25,61]. In this study, the analyses of heavy metal contamination in pond sediments showed similar contamination from fish feed, in both pangasius and tilapia ponds. Accumulation of heavy metals in sediments could be a major source of contamination for fish, since sediment quality affects the feeding habitat, i.e. the bottom of fish ponds [22]. This is shown in the fish farmed in the old ponds, which had higher concentrations of heavy metals. Other heavy metals like Cd and Hg were not found at levels which would cause harm.

Water can be a source of bioaccumulation of heavy metals, and other chemicals by fish [64]. In our study, the concentrations of heavy metals in the water, of both pangasius and tilapia ponds, were lower than the levels in pond sediment and fish feed. These results agree with the study of Aladesanmi et al. [65], who reported that heavy metals accumulate in greater concentrations in sediments, compared to fish tissues and organs, or water, because sediments act as a sink for contaminants. Fish gills are an important site for the uptake of heavy metals because water enters the mouth and is passed across the gill to take the DO from water [22]. Another possible source of entry is fish skin, which is often covered by a layer of mucus. Contact with heavy metals in the water can be absorbed onto the mucus through direct contact with the surrounding water [66].

### 4.3 Risk assessment on human health

The EDI of heavy metals in the fish samples in this study were below the recommended limit, indicating no risk to human health associated with the consumption of pangasius and tilapia from the Mymensingh region of Bangladesh. Similarly, the result of individual and combined THQs of each metal was less than one, suggesting that consumers would not experience significant health risks due to the intake of heavy metals. The estimated lifetime cancer risk (R) of individual heavy metal is lower than 1 × 10^−4^ to 1 × 10^-6^, therefore the consumption of these fish does not pose a considerable health hazard under the current scenario of body weight and consumption. However, the R_T_ (2.42 × 10^-6^ and 1.15 × 10^-6^ for pangasius and tilapia, respectively), the total R was within the range of potential carcinogenic risk (1 × 10^-4^ to 1 × 10^-6^), as recommended by the [40,67], indicating continuous consumption over a long time period (∼70 years) has a potential carcinogenic effect on human. Similar results were reported by Ahmed et al. [56] and Ullah et al. [44] for farmed pangasius and tilapia in Bangladesh. Therefore, the potential health risk to consumers by heavy metal exposure, through farmed fish consumption cannot be ignored. Our study has some limitations because the results have been derived from single fish sampled from each pond, and from the analysis of raw pangasius and tilapia flesh. The chemistry of water and soil varies from one pond to another which may affect the ANCs concentrations in fish flesh. On the other hand, the chemistry of water and soil in different places in a pond may not vary because same feed, drugs, chemicals and irrigated water were applied in the pond. For this reason, we assumed that there is limited scope of variations for ANCs among fish produced within same pond but it needs further analysis for better understanding. According to Sharafi et al. [55] cooking process of food items like rice reduced the concentration of heavy metals significantly compared to raw rice. Cooking process can reduce Pb concentrations in rice by 26 %, and in fish by 19–26 % [55,68]. In our study, Pb concentrations ranged between 1.30–1.95 ppm in fish flesh which are above the permissible limit. As per literature, if the cooking process reduces Pb concentrations by 26 %, fish will be remained unsafe for human consumption but it needs to be proved by further research investigation. Thus, the concerns regarding heavy metals and their adverse eﬀ ;ects on human health, are increasing both locally and globally, therefore, the impact of these pollutants in aquaculture must be urgently investigated in a temporal scale, in broader geographical areas. Furthermore, background information on the consumption of heavy metals by pangasius and tilapia must be established and improved through a monitoring and management framework.

## 5 Conclusion

In this study, residues of ANCs, the use of which are prohibited in fish farming in Bangladesh, were analyzed in pangasius and tilapia samples from 30 individual fish ponds. According to the results of this study, the residues of prohibited ANCs, pesticides and dyes were not found in pangasius and tilapia fish. However, fish flesh was contaminated by heavy metals, particularly Pb and Cr. The fish feeds, both commercial and farm-made, were highly contaminated by Cd, Pb, Cr, Zn and As. Farm-made feeds had higher levels of contamination than commercial feeds. The pond environments (water and sediment) were also contaminated by heavy metals at different concentrations. Some heavy metals particularly Pb and Cr were found in pangasius and tilapia flesh at levels much higher than the permissible limits, which pose potential risks to human health. Many contaminants in farmed fish were reported to vary depending on the species, seasonality, geographical locations and methods of production [69] which need to be researched extensively, and set under revised regulatory exposure limits as proposed by Kostoff et al. [42]. Therefore, a background information system on the ingestion of heavy metals and other contaminants by fish produced through commercial aquaculture in Bangladesh, must be established for the regulatory authorities, in order to provide a monitoring and operational framework of aquaculture farms.

## CRediT authorship contribution statement

**Md. Mehedi Alam:** Conceptualization, Methodology, Data curation, Formal analysis, Writing - original draft, Writing - review & editing. **Mohammad Mahfujul Haque:** Conceptualization, Methodology, Project administration, Investigation, Writing - review & editing, Supervision.

## Declaration of Competing Interest

The authors report no declarations of interest.

## Footnotes

1 Commercial brand feed refers to feed manufactured by large companies and marketed widely throughout the country.

2 Local brand feed is manufactured by smaller companies that supply feed locally.

3 Farm-made feed refers feed produced by the farmers using a small-scale pellet machine on the farm premises.
